# One step, two functions: the mechanism of the ABCH class of transporter serving barrier construction and detoxification in insects

**DOI:** 10.1093/procel/pwaf041

**Published:** 2025-05-18

**Authors:** Bernard Moussian

**Affiliations:** Institut Sophia Agrobiotech, Université Côte d’Azur, INRAe, CNRS, Sophia Antipolis, France

**Figure 1.**
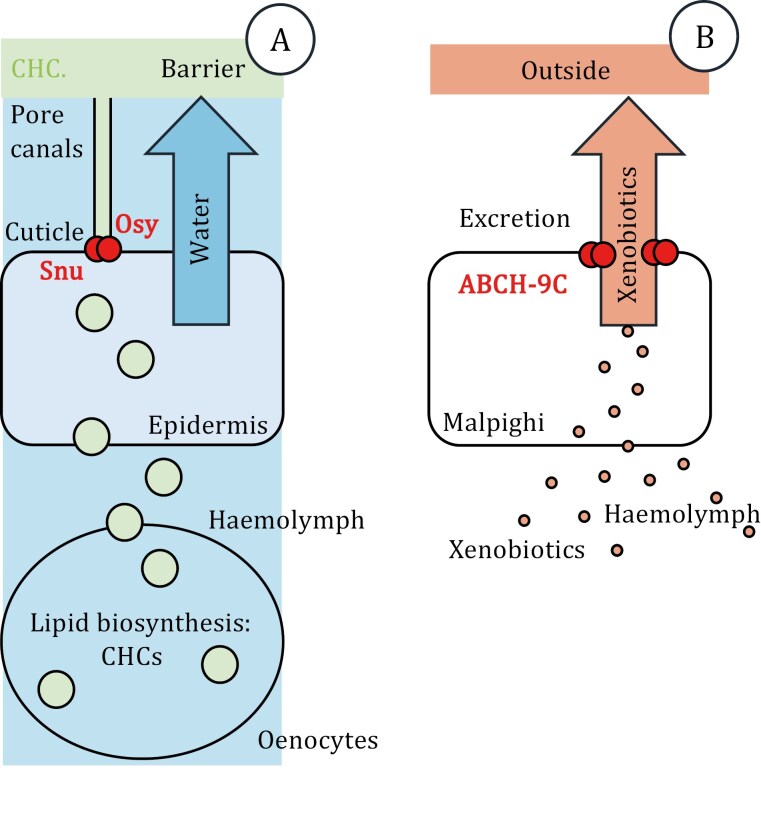
The two roles of ABCH transporters in insects. (A) As demonstrated in the fruit fly *Drosophila melanogaster*, the two ABCH transporters Osy and Snu are involved in cuticular hydrocarbon (CHC, produced in sub-epidermal oenocytes) delivery through the pore canals to the body surface to constitute a water-proof barrier. (B) Xenobiotics accumulating inside the body and inside the cells of different tissues are transported to the lumen of excretory organs such as the Malpighian tubules (insect kidneys) or the hindgut for detoxification.

Insects occupy a broad range of habitats after terrestrialization and radiation from marine crustaceans 519 million years ago (Schwentner et al., 2017). They found themselves in a new environment, foremost jeopardizing them with low humidity, i.e., dry or desiccation conditions. In addition, their co-evolution with land plants both as beneficial and maleficial companions exposed them to plant-produced xenobiotics (secondary metabolites) that as the plant defence molecules may have potential adverse effects on insects. Insects learned to cope with both situations, among others, through the evolution and activation of H-type ATP-binding cassette (ABC) transporters (Fig. 1). Indeed, ABCH transporters are arthropod-specific ABC transporters that, in all kingdoms of living organisms, using ATP hydrolysis, transport molecules from one side of a membrane to the other, thereby influencing the physiological milieus of both compartments.

ABCH transporters have been reported in various insect species to be important against desiccation. In the fruit fly *Drosophila melanogaster*, two ABCH transporters Snustorr (Snu) and Oskyddad (Osy) are essential for the construction of a cuticular surface barrier. This barrier consists of hydrocarbons (termed cuticular hydrocarbons, CHCs) that are transferred to the surface via pore and wax canals, which emanate from the apical plasma membrane of epidermal cells and run as nanotubes through the extracellular cuticle. CHCs are a blend of saturated, unsaturated and branched hydrocarbons with a chain length between 20 and 50 carbon residues that varies depending on the stage of the insect and the species. Mutations in the genes coding for these transporters, *snu* and *osy*, are embryonic and larval lethal in *D*. *melanogaster* (Wang et al., 2020; Zuber et al., 2018). This underlines that these transporters are essential. Their envelope, the outermost cuticle layer that harbours the CHCs is disintegrated probably accounting for the desiccation phenotype of the respective larvae. The CHC levels of flies with suppressed Snu or Osy function, in addition, are reduced. Moreover, Osy was found to be located in pore canals suggesting that CHC transport through the cuticle requires ABCH activity. Knock-down of the Snu ortholog in the pest migratory locust LmABCH-9C by RNA interference has a similar effect on CHC levels, survival and desiccation phenotype (Yu et al., 2017). Likewise, reduction of transcript levels of the Snu ortholog from the storage pest *Tribolium castaneum* enhances desiccation susceptibility (Broehan et al., 2013). Taken together, as *D. melanogaster*, *T. castaneum* and *L. migratoria* represent distantly related insect clades, ABCH transporters reflect a commonly shared evolutionary adaptation of insects to dry conditions by mediating CHC deposition needed for lipid barrier construction on the cuticle surface.

The second challenge that insects encountered during their evolution, i.e., detoxification against plant secondary metabolites was seemingly also accompanied by the evolution of ABCH transporters. In *T. castaneum* and the small brown planthopper, *Laodelphax striatellus* up-regulate the expression of ABCH coding genes when exposed to xenobiotics such as diflubenzuron or chlorpyrifos suggesting their involvement during xenobiotic detoxification by lowering their concentration within the cell (Rösner et al., 2021; Sun et al., 2017). The actual tissue(s) responding to xenobiotic exposure was not identified; indeed, the role of ABCH transporters during detoxification and their position in the underlying pathways has not been studied in detail.

**Figure 2.**
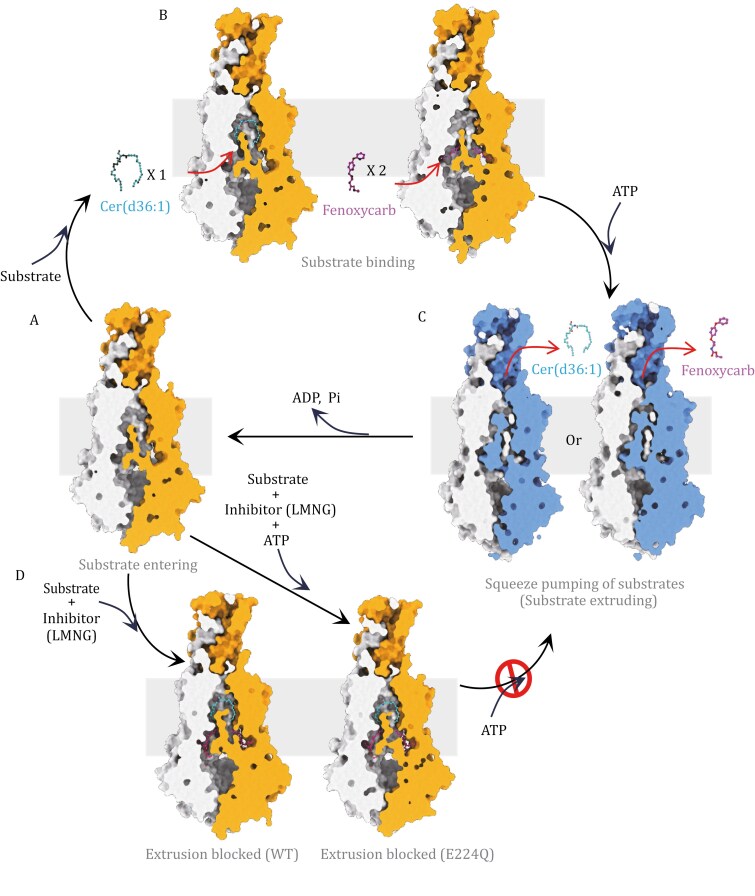
The squeeze-pumping mechanism of cargo transport of ABCH transporters. (A) Substate entering state: A substrate is entering from the cytoplasmic entry site. (B) Substrate binding states: One long-branched-chain substrate represented by ceramide (left) or two short-chain substrates represented by two fenoxycarb molecules (right) are bound to the long, and arched tunnel. (C) Substrate extruding states: A substrate is extruded upon ATP binding by a squeeze pump mechanism. The TMDs squeeze to pump out the bound substrate, ceramide or fenoxycarb, and the arched tunnel is crushed. (D) Substrate extrusion blocked states: Substrate extrusion is suspended by LMNG binding, keeping the substrate-binding tunnel in an open and ATP-free conformation.

Recently, Jinli Chen, Yanwei Duan, Yuanyuan Zhou and Qing Yang published a landmark article in *Cell* that unravels the mechanism of ABCH transporter function in transporting molecules across membranes (Chen et al., 2025). Using cryo-electron microscopy (cryo-EM), they elegantly showed that ceramide and the insecticide fenoxycarb are transported by the TcABCH-9C transporter applying a squeeze-pump mechanism (Fig. 2). As a half transporter, TcABCH-9C homo-dimerizes to constitute a full transporter forming a narrow and arched substrate-binding tunnel that is long enough to harbour lipids such as ceramide or two short lipophilic molecules such as fenoxycarb. Upon binding of the substrate on the cytosolic side of the membrane and ATP binding at the nucleotide-binding domain of the protein, the tunnel crushes and the substrate is squeezed to the vent at the other side of the membrane and pumped into the target compartment. This squeeze-pump mechanism is novel for ABC transporters and possibly unique for insect ABCH transporters indicating the evolution of it in conjunction with the double need for the transport of molecules against desiccation and for xenobiotic tolerance. Excitingly, the authors found that the amphiphilic detergent lauryl maltose neopentyl glycol (LMNG) is able to attenuate TcABCH-9C activity in liposomes by inhibiting both ATPase activity and, by consequence, substrate transport. This landmark finding opens the avenue for the development of a new class of LMNG-based insecticides that should be selective for insect ABCH transporters and therefore environmentally friendly. This is urgently needed as with climate change and its effects on biodiversity dynamics, we require a more sophisticated approach to meet human society and environment with more respect and care. In summary, the detailed elucidation of the mechanism of TcABCH-9C function is, hence, a milestone not only in the cell biology of ABC transporters in general but particularly in the research field of pest management in agriculture.
